# Supplementary material for: Plasma generated ozone and reactive oxygen species for point of use PPE decontamination system
Source: PLoS One. 2022 Feb 25;17(2):e0262818. doi: 10.1371/journal.pone.0262818 (PMC8880944; doi:10.1371/journal.pone.0262818)
Supplement: S1 Table — (DOCX) [file pone.0262818.s001.docx]

S1 Table. Replicates number for each material characterization method

|  | Internal Mechanical Testing | External Strap Tensile Testing | External Filtration Testing | Particulate Filtration Testing | Surface charge | Wettability | Yellowness Index | Hydrostatic Testing | Water Impact Penetration |
| --- | --- | --- | --- | --- | --- | --- | --- | --- | --- |
| Replicate Number | 3 | 3 | 3 | 3 | 6 | 5-6 | 2/6 | 3 | 1 |
