# Supplementary material for: Plasma generated ozone and reactive oxygen species for point of use PPE decontamination system
Source: PLoS One. 2022 Feb 25;17(2):e0262818. doi: 10.1371/journal.pone.0262818 (PMC8880944; doi:10.1371/journal.pone.0262818)
Supplement: S2 Table — (DOCX) [file pone.0262818.s002.docx]

S2 Table. Internal Tensile Testing for Polypropylene

| Polypropylene | | | |
| --- | --- | --- | --- |
| Condition (ppm-min) | Force at Break [N] | | |
| Control-0 | 112.5 | 108 | 108 |
| Ozone 1-700 | 131.8 | 126.4 | 126.4 |
| Ozone 2-1200 | 114.6 | 115.1 | 115.1 |
| Ozone 3-7000 | 107.4 | 116.4 | 116.4 |
|  | Displacement at Break [mm] | | |
| Control-0 | 18.858 | 18.533 | 17.236 |
| Ozone 1-700 | 15.738 | 14.559 | 16.334 |
| Ozone 2-1200 | 19.681 | 20.256 | 17.910 |
| Ozone 3-7000 | 19.737 | 19.161 | 18.795 |
|  | Apparent elongation at Break [%] | | |
| Control-0 | 58.023 | 57.025 | 53.034 |
| Ozone 1-700 | 48.424 | 44.798 | 50.259 |
| Ozone 2-1200 | 60.558 | 62.326 | 55.107 |
| Ozone 3-7000 | 60.728 | 58.958 | 57.829 |
|  |  |  |  |
| Note: Distance between grips = 32.5  Apparent elongation: (displacement/distance between grips) *100 | | | |
