# Supplementary material for: Plasma generated ozone and reactive oxygen species for point of use PPE decontamination system
Source: PLoS One. 2022 Feb 25;17(2):e0262818. doi: 10.1371/journal.pone.0262818 (PMC8880944; doi:10.1371/journal.pone.0262818)
Supplement: S3 Table — (DOCX) [file pone.0262818.s003.docx]

S3 Table. Internal Tensile Testing for Polyester

| Polyester | | | |
| --- | --- | --- | --- |
| Condition (ppm-min) | Force at Break [N] | | |
| Control-0 | 94.9 | 104.8 | 85.9 |
| Ozone 1-700 | 103.8 | 95.4 | 107.2 |
| Ozone 2-1200 | 85.1 | 89.2 | 94.3 |
| Ozone 3-7000 | 99.3 | 99.3 | 105.1 |
|  | Displacement at Break [mm] | | |
| Control-0 | 5.755 | 6.473 | 4.416 |
| Ozone 1-700 | 8.226 | 7.378 | 7.384 |
| Ozone 2-1200 | 4.733 | 5.573 | 6.348 |
| Ozone 3-7000 | 7.086 | 7.128 | 6.493 |
|  | Apparent elongation at Break [%] | | |
| Control-0 | 17.707 | 19.917 | 13.589 |
| Ozone 1-700 | 25.312 | 22.701 | 22.721 |
| Ozone 2-1200 | 14.564 | 17.147 | 19.531 |
| Ozone 3-7000 | 21.804 | 21.934 | 19.980 |
| Note: Gage length = 25 mm  Distance between grips = 32.5 mm  Apparent elongation: (displacement/distance between grips) *100 | | | |
