# Supplementary material for: Plasma generated ozone and reactive oxygen species for point of use PPE decontamination system
Source: PLoS One. 2022 Feb 25;17(2):e0262818. doi: 10.1371/journal.pone.0262818 (PMC8880944; doi:10.1371/journal.pone.0262818)
Supplement: S4 Table — (DOCX) [file pone.0262818.s004.docx]

S4 Table. Internal Tensile Testing for Proxima Gown

| Proxima Gown | | | |
| --- | --- | --- | --- |
| Condition (ppm-min) | Force at Break [N] | | |
| Control-0 | 34 | 31.4 | 32.8 |
| OZONE 1-1800 | 30 | 29.4 | 30.7 |
| OZONE 2-3700 | 29.2 | 31.2 | 34.8 |
|  | Displacement at Break [mm] | | |
| Control-0 | 24.814 | 25.813 | 24.313 |
| OZONE 1-1800 | 22.808 | 20.314 | 21.314 |
| OZONE 2-3700 | 24.314 | 23.814 | 24.314 |
|  | Apparent elongation at Break [%] | | |
| Control-0 | 24.814 | 25.813 | 24.313 |
| OZONE 1-1800 | 22.808 | 20.314 | 21.314 |
| OZONE 2-3700 | 24.314 | 23.814 | 24.314 |
| Note: Distance between grips = 100 mm  Apparent elongation: (displacement/distance between grips) *100 | | | |
