# Supplementary material for: Plasma generated ozone and reactive oxygen species for point of use PPE decontamination system
Source: PLoS One. 2022 Feb 25;17(2):e0262818. doi: 10.1371/journal.pone.0262818 (PMC8880944; doi:10.1371/journal.pone.0262818)
Supplement: S5 Table — (DOCX) [file pone.0262818.s005.docx]

S5 Table. Internal Tensile Testing for BYD three layers and band

| Inner Layer | | | |
| --- | --- | --- | --- |
| Condition (ppm-min) | Force at Break [N] | | |
|  | Replicate-1 | Replicate-2 | Replicate-3 |
| Control-0 | 24.4 | 23.3 | 25.3 |
| Trailer-500 | 23.3 | 23.7 | 23.2 |
| Trailer-1500 | 24.6 | 27.3 | 26.3 |
| Glovebox-500 | 23.6 | 23.7 | 26.9 |
| Glovebox-1500 | 25.6 | 26.2 | 25.1 |
|  | Displacement at Break [mm] | | |
|  | Replicate-1 | Replicate-2 | Replicate-3 |
| Control-0 | 27.929 | 21.599 | 29.100 |
| Trailer-500 | 38.934 | 35.767 | 34.600 |
| Trailer-1500 | 41.600 | 43.600 | 44.600 |
| Glovebox-500 | 23.099 | 21.433 | 34.600 |
| Glovebox-1500 | 30.766 | 27.933 | 25.767 |
|  | Elongation at break [%] | | |
|  | Replicate-1 | Replicate-2 | Replicate-3 |
| Control-0 | 85.935 | 66.460 | 89.537 |
| Trailer-500 | 119.796 | 110.051 | 106.462 |
| Trailer-1500 | 128.000 | 134.154 | 137.231 |
| Glovebox-500 | 71.075 | 65.949 | 106.462 |
| Glovebox-1500 | 94.666 | 85.947 | 79.282 |
| Middle Layer (Cotton) | | | |
| Condition (ppm-min) | Force at Break [N] | | |
|  | Replicate-1 | Replicate-2 | Replicate-3 |
| Control-0 | 10.2 | 11.6 | 11.4 |
| Trailer-500 | 9.2 | 9 | 7.6 |
| Trailer-1500 | 9.4 | 9.7 | 9.4 |
| Glovebox-500 | 10.6 | 10.7 | 10.3 |
| Glovebox-1500 | 9 | 8 | 9.2 |
|  | Displacement at Break [mm] | | |
|  | Replicate-1 | Replicate-2 | Replicate-3 |
| Control-0 | 18.430 | 20.266 | 19.766 |
| Trailer-500 | 20.100 | 16.600 | 15.933 |
| Trailer-1500 | 16.933 | 16.434 | 17.767 |
| Glovebox-500 | 22.933 | 19.767 | 18.434 |
| Glovebox-1500 | 18.434 | 15.267 | 17.767 |
|  | Elongation at break [%] | | |
|  | Replicate-1 | Replicate-2 | Replicate-3 |
| Control-0 | 56.706 | 62.358 | 60.819 |
| Trailer-500 | 61.845 | 51.078 | 49.025 |
| Trailer-1500 | 52.103 | 50.566 | 54.666 |
| Glovebox-500 | 70.563 | 60.820 | 56.719 |
| Glovebox-1500 | 56.719 | 46.975 | 54.666 |
| Middle Layer (Filter) | | | |
| Condition (ppm-min) | Force at Break [N] | | |
|  | Replicate-1 | Replicate-2 | Replicate-3 |
| Control-0 | 10 | 8 | 7.2 |
| Trailer-500 | 6.7 | 7.1 | 6.4 |
| Trailer-1500 | 5.3 | 5.7 | 4.9 |
| Glovebox-500 | 3.9 | 4.2 | 4.8 |
| Glovebox-1500 | 5.3 | 5.8 | 6.1 |
|  | Displacement at Break [mm] | | |
|  | Replicate-1 | Replicate-2 | Replicate-3 |
| Control-0 | 7.763 | 7.266 | 8.100 |
| Trailer-500 | 4.600 | 5.100 | 4.434 |
| Trailer-1500 | 2.934 | 2.100 | 1.434 |
| Glovebox-500 | 1.767 | 1.600 | 2.433 |
| Glovebox-1500 | 2.934 | 3.933 | 3.434 |
|  | Elongation at break [%] | | |
|  | Replicate-1 | Replicate-2 | Replicate-3 |
| Control-0 | 23.885 | 22.357 | 24.922 |
| Trailer-500 | 14.154 | 15.691 | 13.642 |
| Trailer-1500 | 9.027 | 6.461 | 4.412 |
| Glovebox-500 | 5.436 | 4.923 | 7.485 |
| Glovebox-1500 | 9.027 | 12.101 | 10.565 |
| Outer Layer | | | |
| Condition (ppm-min) | Force at Break [N] | | |
|  | Replicate-1 | Replicate-2 | Replicate-3 |
| Control-0 | 32.6 | 27.4 | 30.1 |
| Trailer-500 | 30.6 | 32 | 32 |
| Trailer-1500 | 32.9 | 31.4 | 32.3 |
| Glovebox-500 | 31.8 | 30.7 | 31 |
| Glovebox-1500 | 31.4 | 30.5 | 27.9 |
|  | Displacement at Break [mm] | | |
|  | Replicate-1 | Replicate-2 | Replicate-3 |
| Control-0 | 36.433 | 33.266 | 37.767 |
| Trailer-500 | 39.434 | 38.600 | 37.267 |
| Trailer-1500 | 38.600 | 42.100 | 39.933 |
| Glovebox-500 | 42.266 | 37.433 | 37.600 |
| Glovebox-1500 | 47.432 | 37.933 | 26.432 |
|  | Elongation at break [%] | | |
|  | Replicate-1 | Replicate-2 | Replicate-3 |
| Control-0 | 112.102 | 102.358 | 116.205 |
| Trailer-500 | 121.335 | 118.770 | 114.666 |
| Trailer-1500 | 118.770 | 129.539 | 122.871 |
| Glovebox-500 | 130.050 | 115.180 | 115.692 |
| Glovebox-1500 | 145.946 | 116.718 | 81.330 |
| Band | | | |
| Condition (ppm-min) | Force at Break [N] | | |
|  | Replicate-1 | Replicate-2 | Replicate-3 |
| Control-0 | 53 | 49.7 | 55.1 |
| Trailer-500 | 48.5 | 48.2 | 50 |
| Trailer-1500 | 52.5 | 43.1 | 44.4 |
| Glovebox-500 | 43.7 | 43.5 | 43.1 |
| Glovebox-1500 | 41.9 | 50.3 | 48 |
|  | Displacement at Break [mm] | | |
|  | Replicate-1 | Replicate-2 | Replicate-3 |
| Control-0 | 250.727 | 202.302 | 207.602 |
| Trailer-500 | 196.360 | 199.910 | 194.668 |
| Trailer-1500 | 181.618 | 206.286 | 220.169 |
| Glovebox-500 | 186.077 | 177.302 | 177.511 |
| Glovebox-1500 | 171.219 | 164.419 | 154.044 |
|  | Elongation at break [%] | | |
|  | Replicate-1 | Replicate-2 | Replicate-3 |
| Control-0 | 385.734 | 311.234 | 319.388 |
| Trailer-500 | 302.093 | 307.554 | 299.489 |
| Trailer-1500 | 279.413 | 317.363 | 338.721 |
| Glovebox-500 | 286.272 | 272.772 | 273.093 |
| Glovebox-1500 | 263.413 | 252.952 | 236.990 |
| Note:  Gage length = 20 mm (layers) or = 25 mm(band)  Distance between grips = 32.5 mm (layer) or =65 mm (band)  Apparent elongation: (displacement/distance between grips) *100 | | | |
