# Supplementary material for: Plasma generated ozone and reactive oxygen species for point of use PPE decontamination system
Source: PLoS One. 2022 Feb 25;17(2):e0262818. doi: 10.1371/journal.pone.0262818 (PMC8880944; doi:10.1371/journal.pone.0262818)
Supplement: S6 Table — (DOCX) [file pone.0262818.s006.docx]

S6 Table. Internal Tensile Testing for Prestige Ameritech Gown

| Prestige Ameritech Gown | | | |
| --- | --- | --- | --- |
| Condition (ppm-min) | Force at Break [N] | | |
|  | Replicate-1 | Replicate-2 | Replicate-3 |
| Control-0 | 19.3 | 20.3 | 20.3 |
| Trailer-500 | 16.6 | 15.5 | 19.1 |
| Trailer-1500 | 15 | 15.7 | 11.9 |
| Glovebox-500 | 14.5 | 15.5 | 13.8 |
| Glovebox-1500 | 13.9 | 14 | 13 |
|  | Displacement at Break [mm] | | |
|  | Replicate-1 | Replicate-2 | Replicate-3 |
| Control-0 | 35.312 | 37.314 | 41.814 |
| Trailer-500 | 72.814 | 76.314 | 47.313 |
| Trailer-1500 | 36.814 | 65.813 | 36.314 |
| Glovebox-500 | 65.313 | 71.314 | 50.314 |
| Glovebox-1500 | 57.814 | 55.313 | 47.814 |
|  | Apparent elongation at Break [%] | | |
|  | Replicate-1 | Replicate-2 | Replicate-3 |
| Control-0 | 35.312 | 37.314 | 41.814 |
| Trailer-500 | 72.814 | 76.314 | 47.313 |
| Trailer-1500 | 36.814 | 65.813 | 36.314 |
| Glovebox-500 | 65.313 | 71.314 | 50.314 |
| Glovebox-1500 | 57.814 | 55.313 | 47.814 |
| Note:  Distance between grips = 100 mm  Apparent elongation: (displacement/distance between grips) *100 | | | |
