# Supplementary material for: Plasma generated ozone and reactive oxygen species for point of use PPE decontamination system
Source: PLoS One. 2022 Feb 25;17(2):e0262818. doi: 10.1371/journal.pone.0262818 (PMC8880944; doi:10.1371/journal.pone.0262818)
Supplement: S7 Table — (DOCX) [file pone.0262818.s007.docx]

S7 Table. Internal Tensile Testing for Prestige Ameritech Mask

| Inner Layer | | | |
| --- | --- | --- | --- |
| Condition (ppm-min) | Force at Break [N] | | |
|  | Replicate-1 | Replicate-2 | Replicate-3 |
| Control-0 | 13.2 | 13.3 | 12.9 |
| Trailer-500 | 10 | 10.8 | 11.2 |
| Trailer-1500 | 14.7 | 13.2 | 13.4 |
| Glovebox-500 | 9.9 | 11.3 | 8.6 |
| Glovebox-1500 | 12.2 | 9.1 | 11.5 |
|  | Displacement at Break [mm] | | |
|  | Replicate-1 | Replicate-2 | Replicate-3 |
| Control-0 | 14.433 | 14.100 | 14.767 |
| Trailer-500 | 9.434 | 11.267 | 12.599 |
| Trailer-1500 | 17.267 | 12.267 | 16.266 |
| Glovebox-500 | 13.434 | 12.767 | 6.766 |
| Glovebox-1500 | 15.267 | 8.767 | 10.101 |
|  | Elongation at break [%] | | |
|  | Replicate-1 | Replicate-2 | Replicate-3 |
| Control-0 | 44.408 | 43.383 | 45.435 |
| Trailer-500 | 29.026 | 34.667 | 38.766 |
| Trailer-1500 | 53.129 | 37.744 | 50.050 |
| Glovebox-500 | 41.334 | 39.282 | 20.819 |
| Glovebox-1500 | 46.975 | 26.975 | 31.079 |
| Middle Layer | | | |
| Condition (ppm-min) | Force at Break [N] | | |
|  | Replicate-1 | Replicate-2 | Replicate-3 |
| Control-0 | 10.3 | 10.3 | 10.5 |
| Trailer-500 | 10.9 | 10.1 | 11.1 |
| Trailer-1500 | 10.7 | 11.4 | 10.1 |
| Glovebox-500 | 11.3 | 10.8 | 11.6 |
| Glovebox-1500 | 11.6 | 10.8 | 11.5 |
|  | Displacement at Break [mm] | | |
|  | Replicate-1 | Replicate-2 | Replicate-3 |
| Control-0 | 4.100 | 4.933 | 4.933 |
| Trailer-500 | 4.600 | 4.266 | 4.266 |
| Trailer-1500 | 4.600 | 4.767 | 4.434 |
| Glovebox-500 | 5.600 | 4.434 | 6.100 |
| Glovebox-1500 | 6.100 | 3.933 | 6.100 |
|  | Elongation at break [%] | | |
|  | Replicate-1 | Replicate-2 | Replicate-3 |
| Control-0 | 12.616 | 15.179 | 15.180 |
| Trailer-500 | 14.154 | 13.127 | 13.126 |
| Trailer-1500 | 14.154 | 14.666 | 13.642 |
| Glovebox-500 | 17.231 | 13.642 | 18.769 |
| Glovebox-1500 | 18.770 | 12.102 | 18.770 |
| Outer Layer | | | |
| Condition (ppm-min) | Force at Break [N] | | |
|  | Replicate-1 | Replicate-2 | Replicate-3 |
| Control-0 | 19.9 | 21.1 | 20.4 |
| Trailer-500 | 16.4 | 17.4 | 23.6 |
| Trailer-1500 | 16.4 | 17.4 | 23.6 |
| Glovebox-500 | 19.1 | 18.8 | 22.5 |
| Glovebox-1500 | 19.8 | 21.5 | 22.3 |
|  | Displacement at Break [mm] | | |
|  | Replicate-1 | Replicate-2 | Replicate-3 |
| Control-0 | 27.099 | 31.600 | 34.600 |
| Trailer-500 | 31.266 | 35.600 | 26.767 |
| Trailer-1500 | 20.099 | 20.600 | 41.601 |
| Glovebox-500 | 29.267 | 25.933 | 34.100 |
| Glovebox-1500 | 31.266 | 27.766 | 31.266 |
|  | Elongation at break [%] | | |
|  | Replicate-1 | Replicate-2 | Replicate-3 |
| Control-0 | 83.382 | 97.230 | 106.462 |
| Trailer-500 | 96.202 | 109.539 | 82.359 |
| Trailer-1500 | 61.844 | 63.386 | 128.003 |
| Glovebox-500 | 90.051 | 79.794 | 104.922 |
| Glovebox-1500 | 96.202 | 85.434 | 96.203 |
| Band | | | |
| Condition (ppm-min) | Force at Break [N] | | |
|  | Replicate-1 | Replicate-2 | Replicate-3 |
| Control-0 | 35 | 34.4 | 36.5 |
| Trailer-500 | 33.9 | 35.6 | 35 |
| Trailer-1500 | 33 | 41.3 | 35.8 |
| Glovebox-500 | 42.1 | 39.9 | 31.2 |
| Glovebox-1500 | 35.7 | 37.7 | 33.8 |
|  | Displacement at Break [mm] | | |
|  | Replicate-1 | Replicate-2 | Replicate-3 |
| Control-0 | 111.048 | 103.189 | 93.758 |
| Trailer-500 | 100.109 | 111.203 | 103.923 |
| Trailer-1500 | 105.678 | 118.679 | 99.693 |
| Glovebox-500 | 124.074 | 106.919 | 100.523 |
| Glovebox-1500 | 96.988 | 98.844 | 106.669 |
|  | Elongation at break [%] | | |
| Control-0 | 341.686 | 317.503 | 288.486 |
| Trailer-500 | 308.027 | 342.164 | 319.763 |
| Trailer-1500 | 325.162 | 365.166 | 306.748 |
| Glovebox-500 | 381.766 | 328.980 | 309.302 |
| Glovebox-1500 | 298.426 | 304.135 | 328.212 |
| Note:  Distance between grips = 32.5 mm  Apparent elongation: (displacement/distance between grips) *100 | | | |
