# Supplementary material for: Plasma generated ozone and reactive oxygen species for point of use PPE decontamination system
Source: PLoS One. 2022 Feb 25;17(2):e0262818. doi: 10.1371/journal.pone.0262818 (PMC8880944; doi:10.1371/journal.pone.0262818)
Supplement: S8 Table — (DOCX) [file pone.0262818.s008.docx]

S8 Table. Yellowness Index Testing for Polypropylene

| Yellowness Index - Polypropylene | | | | | | |
| --- | --- | --- | --- | --- | --- | --- |
| Frontside | | | | Backside | | |
| Condition (ppm-min) | Control-0 | 700 | 1200 | Control-0 | 700 | 1200 |
| Replicates |  |  |  |  |  |  |
| 1 | 1.520 | 1.643 | 1.763 | 2.193 | 3.309 | 2.436 |
| 2 | 2.303 | 2.605 | 0.670 | 2.738 | 3.193 | 3.054 |
