# Supplementary material for: Plasma generated ozone and reactive oxygen species for point of use PPE decontamination system
Source: PLoS One. 2022 Feb 25;17(2):e0262818. doi: 10.1371/journal.pone.0262818 (PMC8880944; doi:10.1371/journal.pone.0262818)
Supplement: S9 Table — (DOCX) [file pone.0262818.s009.docx]

S9 Table. Yellowness Index Testing for Polyester

| Yellowness Index - Polyester | | | | | | |
| --- | --- | --- | --- | --- | --- | --- |
| Frontside | | | | Backside | | |
| Condition (ppm-min) | Control-0 | 700 | 1200 | Control-0 | 700 | 1200 |
| Replicates |  |  |  |  |  |  |
| 1 | 1.801 | 2.302 | 2.269 | 3.220 | 2.700 | 2.339 |
| 2 | 2.225 | 2.416 | 1.812 | 3.253 | 3.177 | 2.853 |
