# Supplementary material for: Plasma generated ozone and reactive oxygen species for point of use PPE decontamination system
Source: PLoS One. 2022 Feb 25;17(2):e0262818. doi: 10.1371/journal.pone.0262818 (PMC8880944; doi:10.1371/journal.pone.0262818)
Supplement: S10 Table — (DOCX) [file pone.0262818.s010.docx]

S10 Table. Yellowness Index Testing for 3M N95 Respirator

| Yellowness Index – 3M N95 Respirator | | | | | | |
| --- | --- | --- | --- | --- | --- | --- |
| Inside | | | | Outside | | |
| Condition (ppm-min) | Control-0 | 1800 | 3300 | Control-0 | 1800 | 3300 |
| Replicates |  |  |  |  |  |  |
| 1 | 6.039 | 5.341 | 4.422 | 11.912 | 18.351 | 15.301 |
| 2 | 5.159 | 6.944 | 4.140 | 12.922 | 17.838 | 15.459 |
