# Supplementary material for: Plasma generated ozone and reactive oxygen species for point of use PPE decontamination system
Source: PLoS One. 2022 Feb 25;17(2):e0262818. doi: 10.1371/journal.pone.0262818 (PMC8880944; doi:10.1371/journal.pone.0262818)
Supplement: S11 Table — (DOCX) [file pone.0262818.s011.docx]

S11 Table. Yellowness Index Testing for BYD DE2322

| Yellowness Index – BYD DE2322 | | | | | |
| --- | --- | --- | --- | --- | --- |
| Inside Surface | | | | | |
| Condition (ppm-min) | Control-0 | Trailer-500 | Trailer-1500 | Glovebox-1500 | Glovebox-1500 |
| Replicates |  |  |  |  |  |
| 1 | 2.560 | 2.767 | 5.236 | 1.351 | 2.153 |
| 2 | 3.132 | 4.263 | 5.943 | 1.952 | 2.940 |
| 3 | 4.230 | 5.410 | 5.957 | 2.336 | 3.089 |
| 4 | 2.822 | 4.075 | 5.152 | 1.943 | 2.389 |
| 5 | 4.517 | 5.639 | 5.388 | 2.559 | 3.235 |
| 6 | 5.959 | 5.221 | 5.616 | 3.726 | 3.042 |
| 7 | 2.456 | 3.865 | 5.238 | 1.016 | 2.426 |
| 8 | 2.646 | 5.411 | 5.641 | 1.949 | 3.576 |
| 9 | 3.028 | 6.407 | 6.010 | 2.052 | 3.872 |
| 10 | 2.478 | 4.861 | 5.506 | 1.876 | 2.655 |
| 11 | 3.769 | 5.773 | 6.022 | 2.697 | 3.540 |
| 12 | 4.640 | 6.171 | 6.632 | 4.126 | 3.868 |
|  |  |  |  |  |  |
| Outside Surface | | | | | |
| Condition (ppm-min) | Control-0 | Trailer-500 | Trailer-1500 | Glovebox-1500 | Glovebox-1500 |
| Replicates |  |  |  |  |  |
| 1 | -54.264 | -50.280 | -57.076 | -56.158 | -50.151 |
| 2 | -52.150 | -52.661 | -49.205 | -50.936 | -57.864 |
| 3 | -54.470 | -52.542 | -51.181 | -57.282 | -53.626 |
| 4 | -53.347 | -54.783 | -48.363 | -52.465 | -55.927 |
| 5 | -50.296 | -55.673 | -50.924 | -56.478 | -55.246 |
| 6 | -52.391 | -50.059 | -54.069 | -55.133 | -54.632 |
| 7 | -53.738 | -53.595 | -55.845 | -50.664 | -55.606 |
| 8 | -53.367 | -50.613 | -51.756 | -49.568 | -54.684 |
| 9 | -52.320 | -50.112 | -46.724 | -50.991 | -51.470 |
| 10 | -55.921 | -48.951 | -50.292 | -54.955 | -53.869 |
| 11 | -51.088 | -53.155 | -58.572 | -52.556 | -55.649 |
| 12 | -53.081 | -48.762 | -51.985 | -54.881 | -48.720 |
|  |  |  |  |  |  |
| Strap | | | | | |
| Condition (ppm-min) | Control-0 | Trailer-500 | Trailer-1500 | Glovebox-1500 | Glovebox-1500 |
| Replicates |  |  |  |  |  |
| 1 | 3.991 | 6.679 | 5.250 | 7.131 | 3.675 |
| 2 | 8.575 | 5.654 | 7.080 | 9.223 | 6.875 |
| 3 | 6.590 | 8.212 | 4.885 | 5.555 | 7.212 |
| 4 | 6.302 | 3.830 | 0.232 | 6.269 | 2.642 |
| 5 | -0.560 | 4.123 | 4.649 | 6.271 | 4.424 |
| 6 | 4.521 | 9.995 | 5.967 | -3.186 | -4.582 |
| 7 | 8.948 | 7.302 | 8.429 | 4.462 | 1.794 |
| 8 | 8.972 | 4.519 | 6.380 | 3.840 | 1.067 |
| 9 | 6.837 | 6.220 | 6.100 | 5.738 | 6.044 |
| 10 | 8.646 | 6.229 | 4.731 | 7.391 | 4.135 |
| 11 | 8.040 | 10.626 | 1.634 | 2.879 | 3.923 |
| 12 | 3.941 | 8.279 | 4.252 | 3.655 | -2.368 |
