# Supplementary material for: Plasma generated ozone and reactive oxygen species for point of use PPE decontamination system
Source: PLoS One. 2022 Feb 25;17(2):e0262818. doi: 10.1371/journal.pone.0262818 (PMC8880944; doi:10.1371/journal.pone.0262818)
Supplement: S12 Table — (DOCX) [file pone.0262818.s012.docx]

S12 Table. Yellowness Index Testing for Prestige Ameritech Respirator

| Yellowness Index - Prestige Ameritech Respirator | | | | | |
| --- | --- | --- | --- | --- | --- |
| Inside Surface | | | | | |
| Condition (ppm-min) | Control-0 | Trailer-500 | Trailer-1500 | Glovebox-1500 | Glovebox-1500 |
| Replicates |  |  |  |  |  |
| 1 | 4.081 | 4.363 | 4.061 | 3.489 | 4.249 |
| 2 | 4.147 | 3.958 | 4.690 | 3.373 | 4.241 |
| 3 | 4.217 | 3.707 | 4.443 | 3.371 | 3.923 |
| 4 | 4.000 | 3.923 | 4.260 | 4.331 | 3.927 |
| 5 | 3.554 | 3.993 | 4.211 | 4.112 | 3.916 |
| 6 | 3.665 | 4.096 | 4.455 | 4.115 | 3.291 |
| 7 | 3.533 | 3.945 | 4.124 | 1.520 | 4.088 |
| 8 | 3.814 | 3.256 | 4.109 | 2.565 | 4.116 |
| 9 | 4.030 | 3.394 | 4.293 | 3.900 | 3.978 |
| 10 | 3.399 | 3.889 | 4.106 | 2.275 | 3.977 |
| 11 | 3.153 | 3.386 | 3.630 | 3.997 | 3.853 |
| 12 | 2.680 | 4.215 | 4.328 | 4.004 | 2.798 |
| Outside Surface | | | | | |
| Condition (ppm-min) | Control-0 | Trailer-500 | Trailer-1500 | Glovebox-1500 | Glovebox-1500 |
| Replicates |  |  |  |  |  |
| 1 | 2.931 | 5.202 | 2.719 | 3.867 | 2.732 |
| 2 | 3.175 | 4.257 | 1.397 | 3.458 | 3.131 |
| 3 | 2.491 | 4.030 | 2.203 | 3.577 | 3.650 |
| 4 | 4.001 | 5.684 | 5.172 | 4.330 | 4.288 |
| 5 | 3.561 | 4.472 | 4.710 | 3.543 | 4.023 |
| 6 | 3.235 | 4.016 | 4.065 | 3.586 | 3.679 |
| 7 | 2.694 | 4.208 | 1.950 | 2.469 | 2.708 |
| 8 | 2.933 | 3.756 | 2.298 | 2.546 | 2.670 |
| 9 | 1.613 | 3.220 | 1.622 | 2.796 | 2.462 |
| 10 | 3.616 | 4.315 | 4.236 | 3.912 | 4.571 |
| 11 | 3.695 | 3.412 | 4.009 | 4.015 | 3.641 |
| 12 | 3.777 | 3.620 | 3.340 | 3.360 | 3.204 |
| Strap | | | | | |
| Condition (ppm-min) | Control-0 | Trailer-500 | Trailer-1500 | Glovebox-1500 | Glovebox-1500 |
| Replicates |  |  |  |  |  |
| 1 | 10.177 | 8.138 | 8.842 | 7.085 | 7.989 |
| 2 | 9.037 | 8.175 | 9.208 | 7.173 | 8.304 |
| 3 | 8.454 | 8.347 | 9.627 | 8.080 | 8.869 |
| 4 | 9.194 | 9.012 | 10.310 | 8.198 | 8.880 |
| 5 | 10.206 | 8.077 | 10.105 | 10.237 | 9.043 |
| 6 | 11.683 | 8.080 | 11.428 | 9.713 | 10.128 |
| 7 | 8.724 | 7.290 | 7.751 | 6.512 | 7.479 |
| 8 | 8.838 | 7.729 | 8.078 | 6.619 | 8.039 |
| 9 | 8.675 | 6.541 | 8.151 | 6.790 | 8.134 |
| 10 | 8.660 | 7.469 | 8.428 | 8.219 | 8.441 |
| 11 | 10.681 | 7.698 | 8.835 | 8.867 | 8.724 |
| 12 | 12.354 | 7.057 | 9.908 | 9.185 | 8.827 |
