# Supplementary material for: Plasma generated ozone and reactive oxygen species for point of use PPE decontamination system
Source: PLoS One. 2022 Feb 25;17(2):e0262818. doi: 10.1371/journal.pone.0262818 (PMC8880944; doi:10.1371/journal.pone.0262818)
Supplement: S13 Table — (DOCX) [file pone.0262818.s013.docx]

S13 Table. Yellowness Index Testing for Prestige Ameritech Gown

| Yellowness Index - Prestige Ameritech Gown | | | | | |
| --- | --- | --- | --- | --- | --- |
| Condition (ppm-min) | Control-0 | Trailer-500 | Trailer-1500 | Glovebox-1500 | Glovebox-1500 |
| Replicates |  |  |  |  |  |
| 1 | 1.650 | 0.870 | 1.233 | 1.458 | 2.157 |
| 2 | 1.717 | 1.063 | 1.302 | 1.686 | 2.049 |
| 3 | 1.168 | 0.961 | 0.979 | 1.548 | 1.629 |
| 4 | 1.393 | 1.361 | 2.276 | 0.930 | 1.729 |
| 5 | 1.558 | 1.260 | 1.725 | 0.918 | 2.016 |
| 6 | 2.012 | 1.181 | 1.231 | 1.599 | 1.723 |
| 7 | 1.532 | 0.229 | 1.293 | 1.584 | 2.150 |
| 8 | 1.714 | 0.453 | 1.426 | 1.606 | 1.830 |
| 9 | 1.446 | 0.758 | 0.673 | 1.497 | 1.522 |
| 10 | 1.444 | 0.765 | 1.240 | 1.828 | 1.698 |
| 11 | 1.379 | 1.058 | 1.510 | 1.484 | 1.494 |
| 12 | 1.697 | 0.735 | 1.254 | 1.875 | 1.656 |
