# Supplementary material for: Plasma generated ozone and reactive oxygen species for point of use PPE decontamination system
Source: PLoS One. 2022 Feb 25;17(2):e0262818. doi: 10.1371/journal.pone.0262818 (PMC8880944; doi:10.1371/journal.pone.0262818)
Supplement: S14 Table — (DOCX) [file pone.0262818.s014.docx]

S14 Table. Surface Wettability Testing for Proxima Gown

| Surface Wettability/Water Contact Angle [°] | | | | | | |
| --- | --- | --- | --- | --- | --- | --- |
|  | Frontside | | | Backside | | |
| Condition (ppm-min) | Control-0 | 1800 | 3700 | Control-0 | 1800 | 3700 |
| Sample# |  |  |  |  |  |  |
| 1 | 150.5 | 143.979 | 148.716 | 143.929 | 140.75 | 126.167 |
| 2 | 143.826 | 148.78 | 127.742 | 148.169 | 142.324 | 137.544 |
| 3 | 143.26 | 148.081 | 136.221 | 137.385 | 151.832 | N/A |
| 4 | 144.477 | 149.14 | 151.791 | 143.919 | 139.992 | 119.512 |
| 5 | 144.287 | 149.096 | 138.304 | 143.956 | 140.054 | 115.095 |
| 6 | 136.348 | 148.56 | 150.188 | 143.566 | N/A | 127.688 |
