# Supplementary material for: Plasma generated ozone and reactive oxygen species for point of use PPE decontamination system
Source: PLoS One. 2022 Feb 25;17(2):e0262818. doi: 10.1371/journal.pone.0262818 (PMC8880944; doi:10.1371/journal.pone.0262818)
Supplement: S15 Table — (DOCX) [file pone.0262818.s015.docx]

S15 Table. Surface Wettability Testing for Polyester

| Surface Wettability/Water Contact Angle [°] | | | | | | | | | | |
| --- | --- | --- | --- | --- | --- | --- | --- | --- | --- | --- |
|  | Frontside | | | | | Backside | | | |  |
| Condition (ppm-min) | Control-0 | 1200 | 3700 | 7000 | | Control-0 | 1800 | 3700 | 7000 |  |
| Replicate |  |  |  | |  |  |  |  |  |  |
| 1 | 128.792 | 121.155 | 102.291 | | 117.455 | 130.131 | 120.256 | 109.156 | 111.13 |  |
| 2 | 125.353 | 118.889 | 101.145 | | 118.13 | 132.851 | 116.628 | 113.76 | 107.06 |  |
| 3 | 129.028 | 128.284 | 116.051 | | 115.458 | 132.826 | 106.8 | 107.397 | 106.188 |  |
| 4 | 129.605 | 114.724 | 108.356 | | 113.388 | 129.692 | 113.731 | 106.197 | 105.031 |  |
| 5 | 123.799 | 119.157 | 104.757 | | 111.911 | 129.453 | 114.564 | 106.13 | 107.094 |  |
| 6 | 125.195 | 115.728 | 105.08 | | 118.993 | 126.96 | 112.314 | 113.184 | 103.187 |  |
