# Supplementary material for: Plasma generated ozone and reactive oxygen species for point of use PPE decontamination system
Source: PLoS One. 2022 Feb 25;17(2):e0262818. doi: 10.1371/journal.pone.0262818 (PMC8880944; doi:10.1371/journal.pone.0262818)
Supplement: S16 Table — (DOCX) [file pone.0262818.s016.docx]

S16 Table. Surface Wettability Testing for Polypropylene

| Surface Wettability/Water Contact Angle [°] | | | | | | | | | | |
| --- | --- | --- | --- | --- | --- | --- | --- | --- | --- | --- |
|  | Frontside | | | | | Backside | | | |  |
| Condition (ppm-min) | Control-0 | 1200 | 3700 | 7000 | | Control-0 | 1800 | 3700 | 7000 |  |
| Replicate |  |  |  | |  |  |  |  |  |  |
| 1 | 118.441 | 116.988 | 106.892 | | 100.838 | 128.984 | 118.313 | 119.289 | 113.447 |  |
| 2 | 118.441 | 115.219 | 104.297 | | 100.098 | 126.967 | 123.069 | 122.261 | 110.237 |  |
| 3 | 117.182 | 115.491 | 109.157 | | 104.381 | 128.009 | 121.986 | 117.6 | 111.982 |  |
| 4 | 117.978 | 115.777 | 111.457 | | 102.818 | 126.86 | 127.137 | 117.447 | 109.286 |  |
| 5 | 116.616 | 118.426 | 110.254 | | 98.593 | 127.931 | 123.267 | 117.233 | 110.034 |  |
