# Supplementary material for: Plasma generated ozone and reactive oxygen species for point of use PPE decontamination system
Source: PLoS One. 2022 Feb 25;17(2):e0262818. doi: 10.1371/journal.pone.0262818 (PMC8880944; doi:10.1371/journal.pone.0262818)
Supplement: S17 Table — (DOCX) [file pone.0262818.s017.docx]

S17 Table. Water Impact Penetration Testing for Proxima Gown and Prestige Ameritech Gown

|  | Proxima Gown | | | Prestige Ameritech Gown | | | | |
| --- | --- | --- | --- | --- | --- | --- | --- | --- |
| Condition (ppm-min) | Control-0 | 1800 | 3700 | Control- 0 | Trailer-500 | Trailer-1500 | Glovebox-500 | Glovebox-500 |
| Water Gain (g) | 0.05 | 0.06 | 0.08 | 0.01 | 0.01 | 0.01 | 0.01 | 0.01 |
