# Supplementary material for: Plasma generated ozone and reactive oxygen species for point of use PPE decontamination system
Source: PLoS One. 2022 Feb 25;17(2):e0262818. doi: 10.1371/journal.pone.0262818 (PMC8880944; doi:10.1371/journal.pone.0262818)
Supplement: S18 Table — (DOCX) [file pone.0262818.s018.docx]

S18 Table. Surface Charge Measurement for Polypropylene

| Surface Charge Measurement [mm] | | | | |
| --- | --- | --- | --- | --- |
|  | Before charging | | | |
| Condition (ppm-min) | Control-0 | 700 | 1200 | 7000 |
| Replicate |  |  |  |  |
| 1 | 3.175 | 19.844 | 21.431 | 14.288 |
| 2 | 5.556 | 22.225 | 27.781 | 12.7 |
| 3 | 3.969 | 19.844 | 21.431 | 7.938 |
| 4 | 5.556 | 31.75 | 26.988 | 23.019 |
| 5 | 6.35 | 24.606 | 26.194 | 20.638 |
| 6 | 11.113 | 23.8125 | 20.638 | 20.638 |
|  | Immediately after charging | | | |
| 1 | 27.781 | 39.688 | 31.75 | 28.575 |
| 2 | 26.988 | 27.781 | 37.306 | 28.575 |
| 3 | 23.812 | 36.512 | 30.163 | 25.4 |
| 4 | 26.194 | 38.898 | 26.988 | 29.369 |
| 5 | 25.796 | 42.069 | 23.812 | 32.544 |
| 6 | 28.575 | 31.75 | 27.781 | 33.338 |
|  | 15 Days after charging | | | |
| 1 | 6.35 | 2.381 | 4.763 | N/A |
| 2 | 7.938 | 1.588 | 7.938 | N/A |
| 3 | 4.763 | 3.175 | N/A | 3.175 |
| 4 | 11.113 | N/A | N/A | 3.175 |
| 5 | 8.731 | 1.588 | N/A | N/A |
| 6 | 4.763 | 3.175 | 7.938 | 7.938 |
