# Supplementary material for: Plasma generated ozone and reactive oxygen species for point of use PPE decontamination system
Source: PLoS One. 2022 Feb 25;17(2):e0262818. doi: 10.1371/journal.pone.0262818 (PMC8880944; doi:10.1371/journal.pone.0262818)
Supplement: S19 Table — (DOCX) [file pone.0262818.s019.docx]

S19 Table. Particulate Filtration Testing for Prestige Ameritech Respirator

| Prestige Ameritech Respirator | | | |
| --- | --- | --- | --- |
| Condition (ppm-min) | Initial Filter Resistance (mmH_2_O) | | |
|  | Replicate-1 | Replicate-2 | Replicate-3 |
| Control-0 | 7.600 | 7.400 | 7.800 |
| Ozone 500 | 7.500 | 7.600 | 7.700 |
| Ozone 1500 | 7.700 | 7.400 | 7.700 |
|  | Particulate filtration Efficiency (%) | | |
|  | Replicate-1 | Replicate-2 | Replicate-3 |
| Control-0 | 99.340 | 97.790 | 98.620 |
| Ozone 500 | 99.520 | 99.560 | 99.350 |
| Ozone 1500 | 99.590 | 99.530 | 99.500 |
