# Supplementary material for: Plasma generated ozone and reactive oxygen species for point of use PPE decontamination system
Source: PLoS One. 2022 Feb 25;17(2):e0262818. doi: 10.1371/journal.pone.0262818 (PMC8880944; doi:10.1371/journal.pone.0262818)
Supplement: S20 Table — (DOCX) [file pone.0262818.s020.docx]

S20 Table. Particulate Filtration Testing for BYD DE2322

| BYD DE2322 | | | |
| --- | --- | --- | --- |
| Condition (ppm-min) | Initial Filter Resistance (mmH_2_O) | | |
|  | Replicate-1 | Replicate-2 | Replicate-3 |
| Control-0 | 14.000 | 13.000 | 14.700 |
| Ozone 500 | 10.800 | 13.500 | 14.400 |
| Ozone 1500 | 15.400 | 13.300 | 14.100 |
|  | Particulate filtration Efficiency (%) | | |
|  | Replicate-1 | Replicate-2 | Replicate-3 |
| Control-0 | 97.630 | 98.470 | 98.250 |
| Ozone 500 | 97.790 | 98.040 | 97.790 |
| Ozone 1500 | 98.690 | 97.100 | 97.760 |
