# Supplementary material for: Plasma generated ozone and reactive oxygen species for point of use PPE decontamination system
Source: PLoS One. 2022 Feb 25;17(2):e0262818. doi: 10.1371/journal.pone.0262818 (PMC8880944; doi:10.1371/journal.pone.0262818)
Supplement: S21 Table — (DOCX) [file pone.0262818.s021.docx]

S21 Table. Strap Tensile Testing for Prestige Ameritech Respirator

| Prestige Ameritech Respirator | | | |
| --- | --- | --- | --- |
| Condition (ppm-min) | Force in Top Strap (N) | | |
|  | Replicate-1 | Replicate-2 | Replicate-3 |
| Control-0 | 2.574 | 2.663 | 2.514 |
| Ozone 500 | 2.553 | 2.693 | 2.832 |
| Ozone 1500 | 2.687 | 2.600 | 2.599 |
|  | Force in Bottom Strap (N) | | |
|  | Replicate-1 | Replicate-2 | Replicate-3 |
| Control-0 | 3.010 | 2.798 | 2.937 |
| Ozone 500 | 2.876 | 2.966 | 3.234 |
| Ozone 1500 | 3.079 | 3.026 | 2.853 |
