# Supplementary material for: Plasma generated ozone and reactive oxygen species for point of use PPE decontamination system
Source: PLoS One. 2022 Feb 25;17(2):e0262818. doi: 10.1371/journal.pone.0262818 (PMC8880944; doi:10.1371/journal.pone.0262818)
Supplement: S22 Table — (DOCX) [file pone.0262818.s022.docx]

S22 Table. Strap Tensile Testing for BYD DE2322

| BYD DE2322 | | | |
| --- | --- | --- | --- |
| Condition (ppm-min) | Force in Top Strap (N) | | |
|  | Replicate-1 | Replicate-2 | Replicate-3 |
| Control-0 | 4.564 | 4.501 | 4.666 |
| Ozone 500 | 4.721 | 4.686 | 4.800 |
| Ozone 1500 | 4.915 | 5.015 | 5.087 |
|  | Force in Bottom Strap (N) | | |
|  | Replicate-1 | Replicate-2 | Replicate-3 |
| Control-0 | 4.628 | 4.736 | 5.005 |
| Ozone 500 | 4.506 | 4.387 | 4.795 |
| Ozone 1500 | 4.492 | 4.485 | 4.693 |
