# Supplementary figures and images for: Plasma generated ozone and reactive oxygen species for point of use PPE decontamination system
Source: PLoS One. 2022 Feb 25;17(2):e0262818. doi: 10.1371/journal.pone.0262818 (PMC8880944; doi:10.1371/journal.pone.0262818)

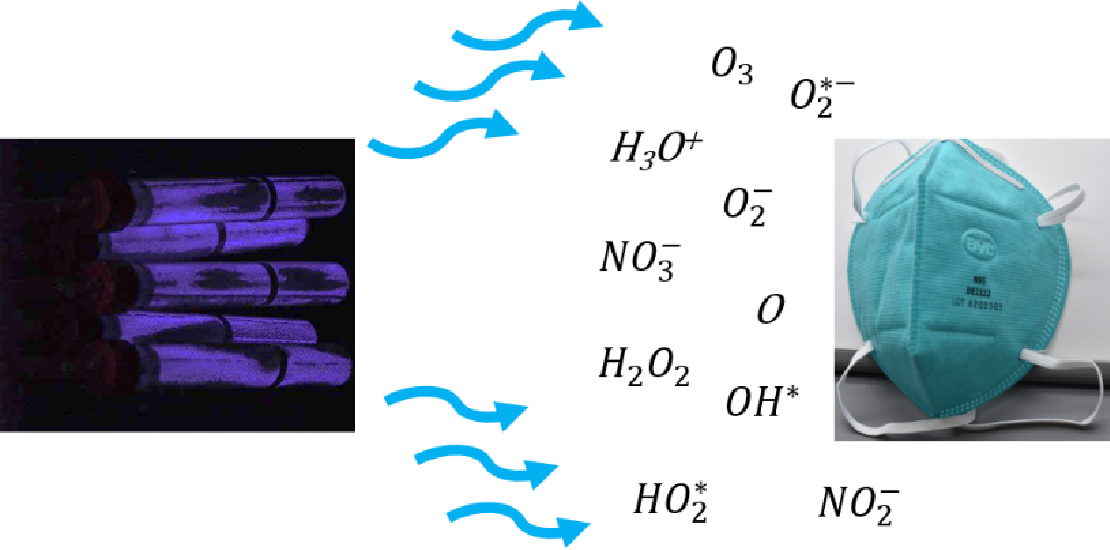

Supplement: S1 Graphical abstract — (TIF) [file pone.0262818.s023.tif]
