# Supplementary material for: Plasma generated ozone and reactive oxygen species for point of use PPE decontamination system
Source: PLoS One. 2022 Feb 25;17(2):e0262818. doi: 10.1371/journal.pone.0262818 (PMC8880944; doi:10.1371/journal.pone.0262818)
Supplement: Attachment — Submitted filename: Responsese to Reviewers_12082021.docx [file pone.0262818.s024.docx]

Subject: Response to review PONE-D-21-23319	Nov. 21, 2021

Dear Editor,

Thank you for the opportunity to revise our paper on ‘*Plasma Generated Ozone and Reactive Oxygen Species for Point of Use PPE Decontamination System*’. The suggestions offered by the reviewers have been immensely helpful, and we also appreciate your interest in publishing the paper in the PLOS ONE.

I have included the reviewer comments immediately after this letter and responded to them individually, indicating exactly how we addressed each concern or problem and describing the changes we have made. The revisions have been approved by all authors. The changes are highlighted in Maroon in the revised paper with track changes as you required, and the unmarked version of the revised manuscript without tracked changes is uploaded through the online system.

We hope the revised manuscript will be acceptable to PLOS ONE and are happy to consider further revisions, and we thank you for your continued interest in our research.

Sincerely yours,

| Dr. David Staack  [dstaack@tamu.edu](mailto:dstaack@tamu.edu)  +1 (979)-845-4063 | Associate Professor, Department of Mechanical Engineering  Texas A&M University, College Station, Texas, USA |
| --- | --- |

1. Is the manuscript technically sound, and do the data support the conclusions?

The manuscript must describe a technically sound piece of scientific research with data that supports the conclusions. Experiments must have been conducted rigorously, with appropriate controls, replication, and sample sizes. The conclusions must be drawn appropriately based on the data presented.

Reviewer #1: Partly

2. Has the statistical analysis been performed appropriately and rigorously?

Reviewer #1: No

3. Have the authors made all data underlying the findings in their manuscript fully available?

The PLOS Data policy requires authors to make all data underlying the findings described in their manuscript fully available without restriction, with rare exception (please refer to the Data Availability Statement in the manuscript PDF file). The data should be provided as part of the manuscript or its supporting information, or deposited to a public repository. For example, in addition to summary statistics, the data points behind means, medians and variance measures should be available. If there are restrictions on publicly sharing data—e.g. participant privacy or use of data from a third party—those must be specified.

Reviewer #1: Yes

4. Is the manuscript presented in an intelligible fashion and written in standard English?

PLOS ONE does not copyedit accepted manuscripts, so the language in submitted articles must be clear, correct, and unambiguous. Any typographical or grammatical errors should be corrected at revision, so please note any specific errors here.

Reviewer #1: Yes

**5. Review Comments to the Author**

Please use the space provided to explain your answers to the questions above. You may also include additional comments for the author, including concerns about dual publication, research ethics, or publication ethics. (Please upload your review as an attachment if it exceeds 20,000 characters)

**Reviewer #1:** This paper reports the DBD plasma reactor-based ozone and reactive oxygen species generation for the decontamination of respirators and gowns. This timely work mainly tested the functionality/stability of the N95 respirators and gowns after 1 and 3 cycles of decontamination in the glovebox and mobile trailer system. However, most of the experiments were conducted with only one sample, this could undermine the validity of the study.

Due to fewer sample size, there was no significant correlation on effect magnitudes of decontamination cycles against different properties tested. Please, provide raw experimental data as supplement information.

The observed structural damage in the brand new PPEs during 3 cycles of decontamination underscores that there may more damage in the used PPEs. Hence, the efficacy of the used PPEs after the first decontamination cycle needs to be tested to validate as a commercially viable technique and the number of recycles that could be possibly achieved in practical remains naïve.

Since this study was designed to disinfect the PPEs using ozone and reuse during this COVID-19 pandemic, the possible dose and mechanism of disinfection of SARS-CoV-2 should be discussed in the review or conclusion section.

As most of the studies, this study also reported only the physical property of the tested material, it is essential to validate remaining gaseous ozone residues and derivatives on the PPEs because these residues/ derivatives on the PPEs may pose inhalation and/or dermal exposure risk.

**Responses to reviewer’s comments:**

Note: Yellow-highlighted is the reviewer’s comments we specifically addressed, and responses are in maroon font color

**Reviewer #1**: This paper reports the DBD plasma reactor-based ozone and reactive oxygen species generation for the decontamination of respirators and gowns. This timely work mainly tested the functionality/stability of the N95 respirators and gowns after 1 and 3 cycles of decontamination in the glovebox and mobile trailer system. However, most of the experiments were conducted with only one sample, this could undermine the validity of the study.

Yes, only a limited number of samples was implemented due to the shortage of the products during the COVID19 pandemic. The number of samples treated is listed in table 4. However, we would like to point out that for each respirator sample sent for external lab testing, 3 replicates were used. For internal material testing a single treated sample was cut into multiple pieces for replication of the material and functionality testing. A paragraph has been added to section 3.3.1 to clarify this.

Due to fewer sample size, there was no significant correlation on effect magnitudes of decontamination cycles against different properties tested. Please, provide raw experimental data as supplement information.

Raw tabulated data on results from individual trials has been provided as supplemental information.

On all plots 2𝜎 is shown, this has been explicitly stated as a general statement about statistics in the new paragraph in section 3.3.1. In some cases, only a single replicate test was performed.

You are correct that for most cases the effect of ozone dose does not cause statistically significant property changes. i.e. we cannot reject the null hypothesis. However, it is not clear that a larger sample size would necessarily lead to a significant correlation with dose (at least over the dose range tested) in all cases. At higher doses, we do expect degradation.

The observed structural damage in the brand-new PPEs during 3 cycles of decontamination underscores that there may more damage in the used PPEs. Hence, the efficacy of the used PPEs after the first decontamination cycle needs to be tested to validate as a commercially viable technique and the number of recycles that could be possibly achieved in practical remains naïve.

You are correct that this testing only takes into account damage to new PPE caused by the decontamination process. There may be other typical use factors that would additionally damage used PPE. A PPE decontamination and reuse protocol would first require inspection of the used PPE to ensure that field use did not damage the PPE. Only PPE which is intact and functional should be subject to a decontamination process. Taking into account both field use and decontamination-induced damage the practical application of PPE recycling is reduced. Testing and decontamination of used PPE were beyond the scope of this project. Cross correlated effects or combined field use damage and decontamination damage are possible but unknown at this time. This stipulation has been added to the ‘conclusion’ section.

Since this study was designed to disinfect the PPEs using ozone and reuse during this COVID-19 pandemic, the possible dose and mechanism of disinfection of SARS-CoV-2 should be discussed in the review or conclusion section.

The possible dose for bioburden reduction and decontamination has been discussed in the literature review section. This does not particularly address SAR-CoV-2 as literature on that is not available yet. However, the applicable mechanism of disinfection is translatable to other viruses like SARS-CoV-2 and additional explanation of this has been added to the ‘literature review’ section, and more detailed information has been discussed in the ‘reaction mechanism’ section:

While SAR-CoV-2 (or any coronavirus) is not specifically addressed by this literature review viruses, similar enveloped viruses presented in Table 1 is Φ6. Vaccinia virus, vesicular stomatitis virus, yellow fever virus, and Sendai virus are all enveloped viruses, and potential explanations for their ozone inactivation are protein capsid damage and genome degradation. There is a great agreement on the alterations, which are induced by ozone in the lipids and proteins present in the membrane of these viruses [1]. As a preferred method used for the generation of ozone, corona discharge also provides another potential mechanism for the decontamination of viruses. Although reactive oxygen and/or nitrogen species (RONS) play the most important role in sterilizing microorganisms, ultraviolet radiation induces direct damage to microorganisms and bacteria via breakage of DNA/RNA and chemical alterations of bases due to the absorption of high energy. More detailed information is covered in section 3.1.2 regarding direct ozone-induced and indirect ozone-generated oxidant species-induced damages. Increasingly attention on ozone inactivation of the enveloped virus is aware, and the mechanism has been discussed in recent researches [2–4]. Research also indicates that induced changes in the viral RNA genome, appearing as aggregation and fusion of influenza viruses (also known as enveloped viruses) were observed via SEM after N2 gas plasma treatment [5].

As most of the studies, this study also reported only the physical property of the tested material, it is essential to validate remaining gaseous ozone residues and derivatives on the PPEs because these residues/ derivatives on the PPEs may pose inhalation and/or dermal exposure risk.

The reviewer is likely referring 1) to residual partial oxidation products and oxidized PPE plastics which may have odor or toxicity and 2) residual ozone on the PPE. Regarding 2: Less than 100 ppb ozone level after treatment in glovebox and trailer systems was required to achieve prior to human touch. Also, the rapid decomposition and vaporization of ozone in appropriate aeration diminish the residue risk. Regarding 1: During the treatment of PPEs, ozone will directly react with organic compounds of the targeted viruses and PPE materials. These new compounds may also be residuals. Although ozone and hydrogen peroxide, which are the possible harmful residues on PPE are detectable based on previous research, proper post-decontamination aeration was also indicated to be helpful with the elimination of residues [6]. The potential skin contact risk caused by residual hydrogen peroxide can also be diminished by increasing aeration time, air exchange rate, and temperature.) A discussion of these potential issues has been added to the ‘conclusion’ section.

Reference

1.	Bayarri B, Cruz-Alcalde A, López-Vinent N, Micó MM, Sans C. Can ozone inactivate SARS-CoV-2? A review of mechanisms and performance on viruses. J Hazard Mater. 2021;415.

2.	Dubuis ME, Racine É, Vyskocil JM, Turgeon N, Tremblay C, Mukawera E, et al. Ozone inactivation of airborne influenza and lack of resistance of respiratory syncytial virus to aerosolization and sampling processes. Vol. 16, PLoS ONE. 2021.

3.	Grignani E, Mansi A, Cabella R, Castellano P, Tirabasso A, Sisto R, et al. Safe and Effective Use of Ozone as Air and Surface Disinfectant in the Conjuncture of Covid-19. Gases. 2020;1(1):19–32.

4.	Blanchard EL, Lawrence JD, Noble JA, Xu M, Joo T, Ng NL, et al. Enveloped Virus Inactivation on Personal Protective Equipment by Exposure to Ozone. medRxiv : the preprint server for health sciences. 2020.

5.	Sakudo A, Shimizu N, Imanishi Y, Ikuta K. N2 gas plasma inactivates influenza virus by inducing changes in viral surface morphology, protein, and genomic RNA. Biomed Res Int. 2013;2013.

6.	Kumkrong P, Scoles L, Brunet Y, Baker S, Mercier PHJ, Poirier D. Evaluation of hydrogen peroxide and ozone residue levels on N95 masks following chemical decontamination. J Hosp Infect [Internet]. 2021;111:117–24. Available from: https://doi.org/10.1016/j.jhin.2021.02.018
